# Increases in soil and woody biomass carbon stocks as a result of rangeland riparian restoration

**DOI:** 10.1186/s13021-020-00150-7

**Published:** 2020-07-31

**Authors:** Virginia Matzek, David Lewis, Anthony O’Geen, Michael Lennox, Sean D. Hogan, Shane T. Feirer, Valerie Eviner, Kenneth W. Tate

**Affiliations:** 1 Santa Clara University, 500 El Camino Real, Santa Clara, CA 95053 USA; 2 UC Cooperative Extension, Novato, CA USA; 3 University of California Davis, Davis, CA USA; 4 Olivet Ranch, Santa Rosa, CA USA; 5 University of California Division of Agriculture and Natural Resources, Davis, CA USA

**Keywords:** California, Carbon storage, Floodplain, Grazing, Mediterranean, Riparian buffer, Restoration, Soil organic matter, Salix, Sequestration

## Abstract

**Background:**

Globally, vegetation in riparian zones is frequently the target of restoration efforts because of its importance in reducing the input of eroded sediment and agricultural nutrient runoff to surface waters. Here we examine the potential of riparian zone restoration to enhance carbon sequestration. We measured soil and woody biomass carbon stocks, as well as soil carbon properties, in a long-term chronosequence of 42 streambank revegetation projects in northern California rangelands, varying in restoration age from 1 to 45 years old.

**Results:**

Where revegetation was successful, we found that soil carbon measured to 50 cm depth increased at a rate of 0.87 Mg C ha^−1^ year^−1^ on the floodplain and 1.12 Mg C ha^−1^ year^−1^ on the upper bank landform. Restored sites also exhibited trends toward increased soil carbon permanence, including an increased C:N ratio and lower fulvic acid: humic acid ratio. Tree and shrub carbon in restored sites was modeled to achieve a 50-year maximum of 187.5 Mg C ha^−1^ in the channel, 279.3 Mg ha^−1^ in the floodplain, and 238.66 Mg ha^−1^ on the upper bank. After 20 years of restoration, the value of this carbon at current per-ton C prices would amount to $US 15,000 per km of restored stream.

**Conclusion:**

We conclude that revegetating rangeland streambanks for erosion control has a substantial additional benefit of mitigating global climate change, and should be considered in carbon accounting and any associated financial compensation mechanisms.

## Introduction

Riparian zones have long been a focus of conservation efforts because they provide unique and important ecosystem services that are vulnerable to degradation. Services provided to agricultural producers by intact or restored riparian vegetation may include increases to pollinator diversity and abundance [1, 2]; improved soil stability and resilience to erosion [3, 4], and flood attenuation [5]. Services to society at large may include reduction of drinking water pollutants such as nitrate [6], phosphate [7], pathogens [8, 9], and pesticides [10]; provision of corridors for wildlife passage [11]; bioenergy and biofuels [12]; habitat improvement for sportfish [13]; and buffering of the local climate via cooler water and air temperatures [14]. Riparian zones are hotspots for the provision of goods and services, especially in arid or seasonally dry climates, and are expected to play a critical role in adaptation to climate change [5, 15–17].

**Fig. 1:**
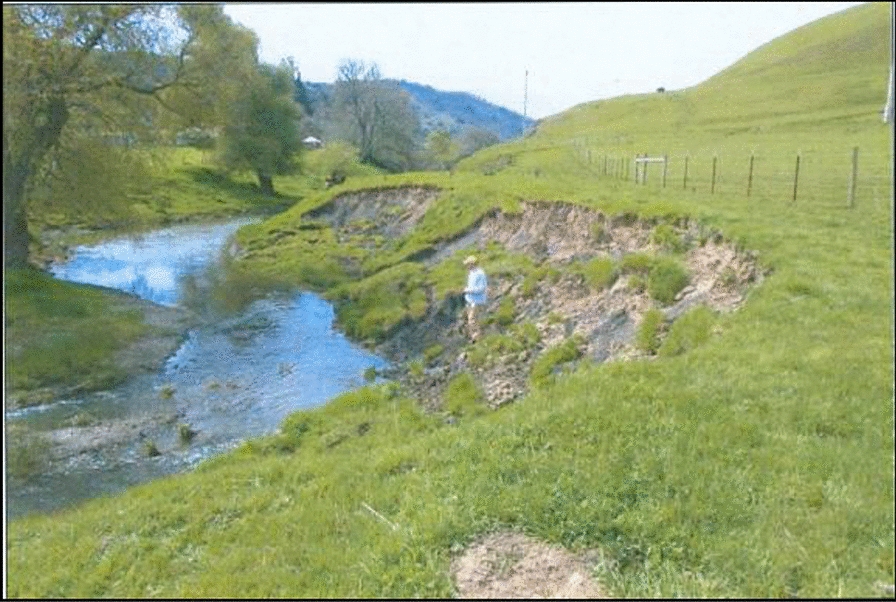
Unvegetated stream bank in Walker Creek, California. Restoration and revegetation projects are designed and implemented in settings like this to reestablish tree and shrubs that protect stream banks from erosion and create wildlife habitat

In our northern California study system, concern about streambank erosion has animated conservation practices for more than half a century, with initial efforts to maintain agricultural productivity gradually coming into alignment with new regulatory mandates to improve water quality and enhance coastal watershed protection [18]. In the 1980s and 1990s, conservation practices such as fencing out livestock from streams, installing biotechnical erosion barriers, and planting native riparian vegetation, gained wide currency in the region as a means of preventing severe topsoil loss and channel incision (Fig. 1). In this paper, we retrospectively examine these projects, which were initially aimed at enhancing local, on-farm soil productivity, for their potential to produce the globally relevant ecosystem service of carbon sequestration.

We anticipated that riparian restoration would increase C sequestration in both the soil and vegetative biomass [19], firstly because the community composition would shift from lower-biomass annual grasslands to higher-biomass forest and shrublands; secondly because the biomass inputs to soil organic matter would have a higher percentage of C when derived from woody plants; and thirdly because preventing erosion would prevent loss of soil C from the riparian zone. We hypothesized that the time since restoration would positively correlate with carbon accumulation; that restored sites would have significantly more soil and biomass carbon than unrestored sites; and that the size of these effects would vary across riparian landforms.

Quantifying the amount of any increased carbon storage from rangeland riparian restoration is particularly relevant to California, where more than half the land surface is rangeland [20], and where some regions have lost as much as 95% of their riparian forest cover [21]. California has a cap-and-trade system for greenhouse gas emissions that includes both the certification of credits for carbon trading with regulated entities such as power plants and utilities, as well as the disbursement of cap-and-trade auction revenues to state agencies that undertake GHG-reducing or -offsetting activities, such as reforestation [22]. Under these programs, the state’s many ranchers and dairy producers could be incentivized to establish more riparian buffers on their lands, as a means of diversifying farm income [23]. We also anticipate that our results could be useful to estimating C storage in other Mediterranean or semi-arid regions with a history of riparian revegetation, such as Australia [24, 25].

## Methods

### Study area

The study region has a Mediterranean climate, with cool wet winters and hot dry summers (MAT = 14.7 °C, MAP = 90 cm). Our project sites were located in watersheds with an average area of 2350 ha (range = 20–3,310 ha), elevation of 145.3 m asl (range = 3.7–656.4 m asl), and forest cover of 21.9% (range = 0–100%). All sites were located on second- and third-order streams within actively grazed rangelands. Rangelands in this region occur on a mosaic of oak woodlands and savannahs, with woodlands having greater than 10% cover of evergreen or deciduous oak tree species, and savannahs dominated by annual grasses with occasional oaks.

**Fig. 2:**
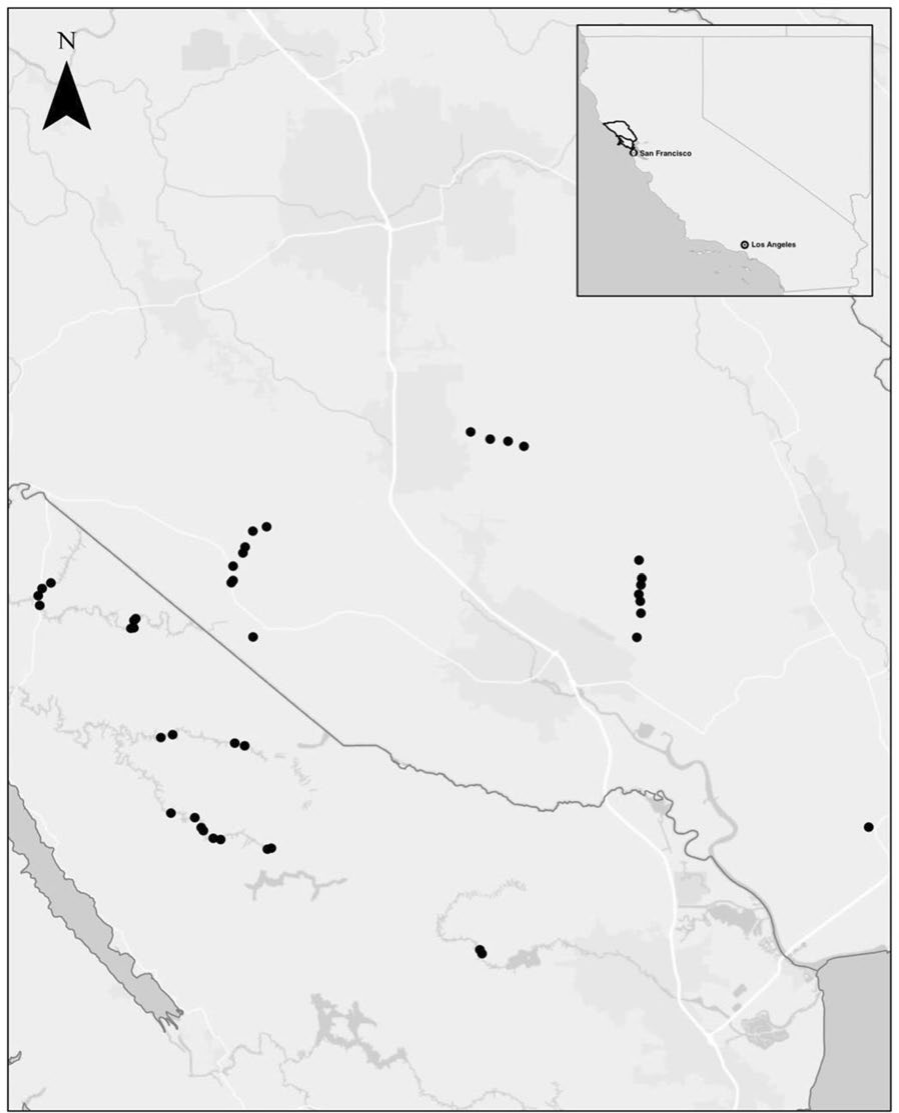
Map of study sites. Dots indicate sites. Inset map shows location of study counties within California

This study employs a retrospective approach to understand the question of multi-decadal carbon accumulation in riparian restoration treatments. While retrospective studies may lack consistency in size or type of treatment, they are often used in managed landscapes to assess temporal changes in long-term processes with greater power and cost-effectiveness than can be achieved through short-term experimentation [26–28]. We examined patterns of carbon sequestration in biomass and soil resulting from riparian restoration in 42 stream reaches in northern California (Fig. 2). Revegetation of riparian zones to achieve erosion control had been attempted on 32 of these reaches, and we included an additional 10 unrestored reaches for comparison. Revegetation projects at these sites were typically installed using a combination approach of three active intervention measures [29], including tree and shrub planting, biotechnical streambank stabilization (e.g., armoring with coarse woody debris), and large herbivore management (e.g., fencing to exclude livestock and/or deer, reduced stocking rate, or removal of grazing). For an analysis focused on the effects of restoration success and time since restoration, we chose riparian revegetation projects ranging in age from 1 to 45 years post-restoration, both successes and failures, that shared a high degree of similarity in geomorphology and landscape setting.

### Soil and plant biomass measurements

**Fig. 3:**
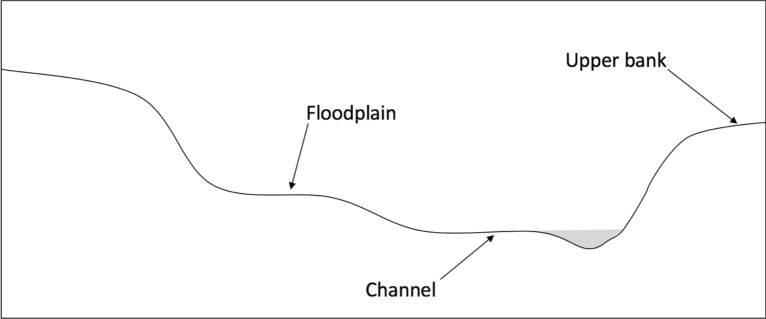
Landscape positions within a stream cross-section. Vegetation and soils were sampled in the active channel (bankfull to water’s edge), floodplain (intermittently flooded terraces at bankfull elevation or above), and on the upper bank (terrace outside the hydrologic floodplain)

At all 42 riparian sites enrolled in this study, field measurements of riparian community composition and biomass were made during summer and early fall 2014 in a single 2-m radial plot placed in the center of a representative section of each of three landforms: active channel, depositional floodplain, and upper bank (Fig. 3), for a total of 126 plots. We defined the active channel as extending from bankfull to the water’s edge at the time of sampling, the depositional floodplain as the first terrace(s) above the active channel that are frequently or infrequently flooded (constituting the remainder of the hydrologic floodplain), and the upper bank as the stream terrace above the hydrologic floodplain. We identified to species and recorded the diameter at breast height (DBH, measured at 1.4 m) of all woody and semi-woody individuals larger than 2.5 cm DBH in each plot. We then used generalized allometric equations [30] to convert DBH of tree and woody shrub species into aboveground biomass. Equation 1 was used for riparian trees (*Salix laevigata, S. lasiolepis, S. lucida, S. exigua, Alnus rhombifolia*) and Eq. 2 for woody shrubs (*Sambucus mexicana, Symphoricarpos alba, Baccharis pilularis*). For semi-woody shrubs like blackberry (*Rubus* spp) and wild rose (*Rosa californica*), we parameterized a simple equation that assumes that DBH and biomass are proportional, based on destructive harvests of *Rubus ursinus* and *Rubus discolor* at our sites, and obtained Eq. 3:

1 $$ {\text{biomass }} = { \exp }( - 2. 20 9 4 { } + \, \left( { 2. 3 8 6 7*{ \ln }\left( {\text{DBH}} \right)} \right) $$

2 $$ {\text{biomass }} = { \exp }( - 0. 7 1 5 2 { } + \, \left( { 1. 70 2 9*{ \ln }\left( {\text{DBH}} \right)} \right) $$

3 $$ {\text{biomass }} = \, 0. 1 7 4 { } \times {\text{ DBH}} $$

Biomass is in kg dry weight and DBH is in centimeters for Eqs. 1–3. A small number (n = 3) of standing dead trees were assigned to decay class 3 [31] and the associated aboveground biomass estimate adjusted downwards using the density reduction factor for *Populus* [32]. Belowground biomass was calculated at the hectare scale using a standard two-parameter equation that predicts root biomass density (RBD, Mg ha^−1^) from age and aboveground biomass (ABG, Mg ha^−1^) for temperate forests [33]; for our region the equation takes the form RBD = exp(−1.3267 + 0.8877*ln(ABG) + .1045*ln(years). In accordance with carbon accounting convention, carbon was assumed to make up 50% of biomass.

Soil sampling was performed at representative locations on each landform at each site: upper bank, floodplain, and active channel (streambed). For floodplain and upper bank locations, we excavated soil pits and collected samples by genetic horizon down to 50 cm for bulk density and laboratory analysis. An additional sample was collected by hand auger to a depth of 100 cm (or to the water table, whichever was shallower). In the active channel, we sampled only the top 10 cm because soils below this depth tended to be dominated by stream sediments (sand, cobbles, gravel) and saturated by the water table. All soil samples were passed through a 2 mm sieve to remove gravel and coarse roots. Bulk density in the top 50 cm was measured by 3-D laser scanning of aggregates [34], except in channel soil samples, from which we could not extract complete peds or accurate volumetric cores. We instead used the bulk density of the top horizon of the adjacent floodplain soil pit as a proxy for the bulk density for channel samples. Soil percentages of carbon (C%) and nitrogen (N%) were analyzed on air-dried, sieved soil samples by a direct combustion analyzer. We verified that the samples contained no detectable carbonates, so C% represents total soil organic C. In order to express soil organic carbon on a dry weight basis, separate subsamples were oven dried at 105 °C for 24 h to correct for the gravimetric water content of air-dried soil. C:N was calculated as an index of soil organic matter (SOM) quality or resistance to decomposition [35]. Two other metrics of SOM quality were determined by chemical fractionation of the organic matter. The labile carbon fraction, a measure of the readily degradable organic residue, was calculated after a permanganate extraction [36, 37]. The fulvic acid: humic acid ratio was calculated by extracting fulvic and humic acids using a NaOH extraction followed by acid precipitation [38]; budgetary constraints precluded performing this analysis on all of our sampled soils, so we randomly chose a subset of 27 sites (2 landforms each, n = 54).

Soil bulk density was used to convert gravimetric measurements to volumetric pools. For channel soil C and N stocks, we performed this calculation on the top 10 cm. However, to avoid confounding changes to organic matter inputs with changes to soil bulk density, for the upper bank and floodplain landforms we calculated soil C and N stocks on an equivalent soil mass basis [39], standardizing on the median soil mass to 50 cm depth, and using the auger sample as necessary to equalize soil mass among profiles. Total soil bulk density, C%, N%, C:N, labile carbon fraction (labile C%), and the fulvic acid: humic acid ratio (FA:HA) were then calculated as mass-weighted averages to 50 cm depth.

**Fig. 4:**
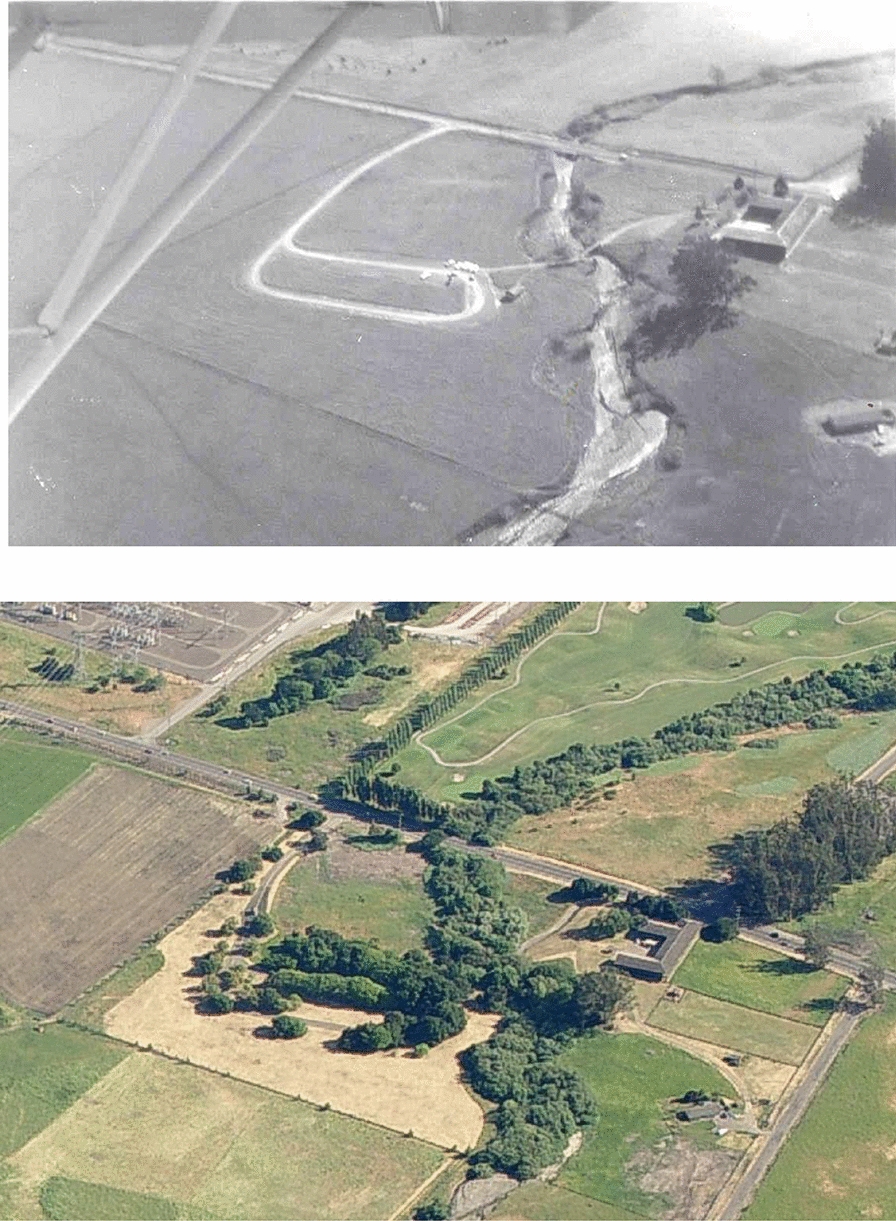
Successful restoration, before and after. Photographs of Adobe Creek prior to restoration in 1971 (top) and 35 years later in 2006 (bottom)

To understand carbon accumulation as a function of time since restoration, we classified sites as “failures” or “successes” based on whether or not any woody riparian vegetation had successfully established at the site (Fig. 4). We then regressed soil carbon and plant biomass carbon on age, with unrestored sites representing an age of zero. Because failed sites would have failed within a year or two of planting, we excluded them from any age-based regression, because we did not consider it appropriate to have a site that had failed as much as four decades earlier figuring into in the regression as a “40-year-old” site. After this adjustment we had a total of 32 sites (n = 96 landforms) for the age-based analyses. We used an ordinary least squares regression model a priori for soil carbon. For plant biomass carbon, we tested linear, quadratic, and logistic growth functions; the best fit with plant biomass carbon was provided by Eq. 4, the logistic growth equation also known as the von Bertalanffy equation:

4 $$ {\text{Biomass}} = a\left( {1 - e^{{ - b\left( {age} \right)}} } \right)^{3} $$

where *a* is the maximum stand-level aboveground tree biomass and *b* is a growth rate that determines how fast the stand reaches its maximum [40]. This is the same methodology used to produce look-up tables for forest biomass in California’s carbon accounting protocol [41]. We used unrestored sites to represent an age of zero years and excluded failed sites from the growth models. Curves were iteratively fit until they converged on an optimal solution, using the nonlinear least squares method in the R package *nlstools* [42]; appropriate starting values were suggested by graphical previews of curve fits. To estimate the propagated error from the nonlinear fit around a single point estimate, we repeated the nonlinear curvefitting in a Bayesian framework in R package *brms* [43], using the parameter estimates for *a* and *b* as priors. We then generated Bayesian 95% confidence intervals from the posterior draws for the point estimate at 20 years of age. For the linear model of soil C accumulation over time, we used the 95% confidence interval for the regression slope coefficient as a measure of the uncertainty around the 20-year point estimate.

In addition to our analysis of carbon pools as a function of restoration age, we also compared floodplain and upper bank soil properties (bulk density, total C and N stocks, C%, N%, C:N, labile C%, and FA:HA) between restored and unrestored sites. We analyzed these data as linear mixed effects models with landform and revegetation status as fixed effects and site as a random effect, using maximum likelihood estimation in R package *lme4* [44]. In this instance we included failed sites in the same category as the unrestored sites, because time since restoration was not a variable in this analysis and because sites with no established vegetation were considered to have continued to erode; this gave us a total of 42 sites (n = 126 landforms). Degrees of freedom and p-values were approximated with the *lmerTest* package [45].

### Spatial analysis and scale-up

The Climate Action Plan for Marin County, where many of our study sites were located, calls for reducing greenhouse gas emissions by an additional 84,160 Mg of carbon dioxide equivalents. To understand the potential role of riparian restoration in meeting this target, we scaled up restored riparian carbon stocks to the whole-county level. We calculated the total restorable area within 3 zones that corresponded to the average widths of the channel, floodplain, and upper bank landforms for a complete stream network within the county’s area of 215,000 hectares. We considered “restorable area” to consist of grassland land cover within 24 m of the stream center, with the 0–3 m zone representing the active channel, the 3–12 m zone representing the floodplain, and the 12–24 m zone representing the upper bank. Elevation and slope layers used to classify the land cover types were derived from LiDAR data collected for all of Marin County in 2010. These layers were spatially resampled and co-georegistered to match a mosaic of National Agricultural Imagery Program (NAIP) layers from 2014, which have a spatial resolution of 1-m ground sample distance. We then combined the elevation-based layers with the NAIP imagery in an 8-layer stack composed of blue, green, red, near infrared, elevation, slope, aspect, and hillshade layers. Using aerial photo interpretation, hundreds of sample areas within the NAIP imagery were identified as one of the five following simplified land cover types: grasslands, shrubs, trees, water/shadows (combined due to spectral similarity), or roads/buildings/spectral glint. Using the layer stack of NAIP imagery and elevational data, we applied a random forests machine learning classifier [46] to the datasets to create a classified layer of the five land cover types.

**Fig. 5:**
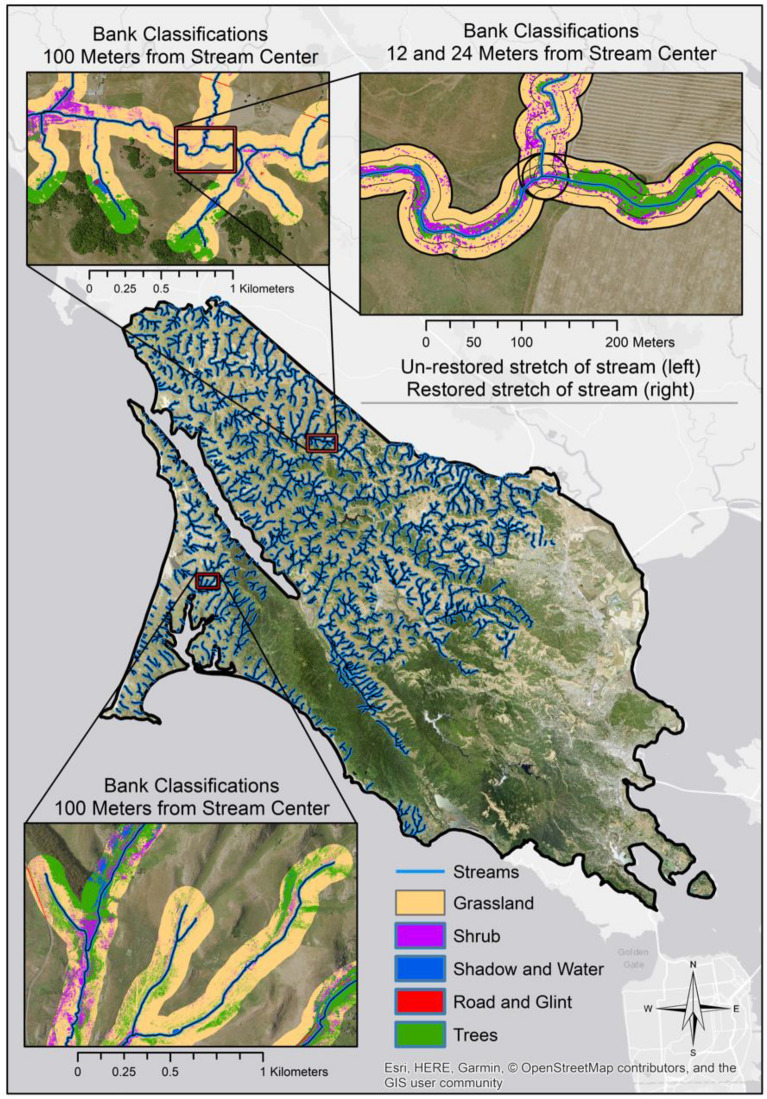
Illustration of Marin County stream network, showing land cover classes mapped within 12-, 24-, and 100-m buffers (insets). The 3-m buffer (not depicted) was used to calculate carbon stocks for the channel landform countywide, while the floodplain was represented by the 3–12 m distance from stream center, and the upper bank represented by the 12–24 m distance

Using a stream flow analysis model applied to the elevation data we created a stream network for the entirety of Marin County (Fig. 5). While directionally accurate in terms of flow, the stream network included substantial spatial errors due to topographic variations that were not detectable by the resolution of the LiDAR scan. Therefore, we used the co-registered 1-m resolution NAIP imagery as a base layer to spatially correct the streamlines to be within less than 3 m of the centers of apparent stream centers, employing aerial photo analysis and re-digitization of the stream network. The total overall stream length was 1602 km, but because streams passing through dense forests or highly urbanized areas were not comparable to our rangeland study sites (nor considered restorable), densely forested and urbanized areas were masked off from the analysis, resulting in a stream network of 1215 km in length. After calculating the total restorable (i.e., grassland) area within each landform, we multiplied it by the total accumulated carbon at 20 years of restoration age. We then adjusted this value to account for the failed sites excluded from the growth curve modeling, by multiplying it by a fraction representing the proportion of successful revegetation attempts on each landform. We calculated this fraction as the ratio of the total area of successful revegetation projects to the total area of attempted revegetation projects.

## Results

**Fig. 6:**
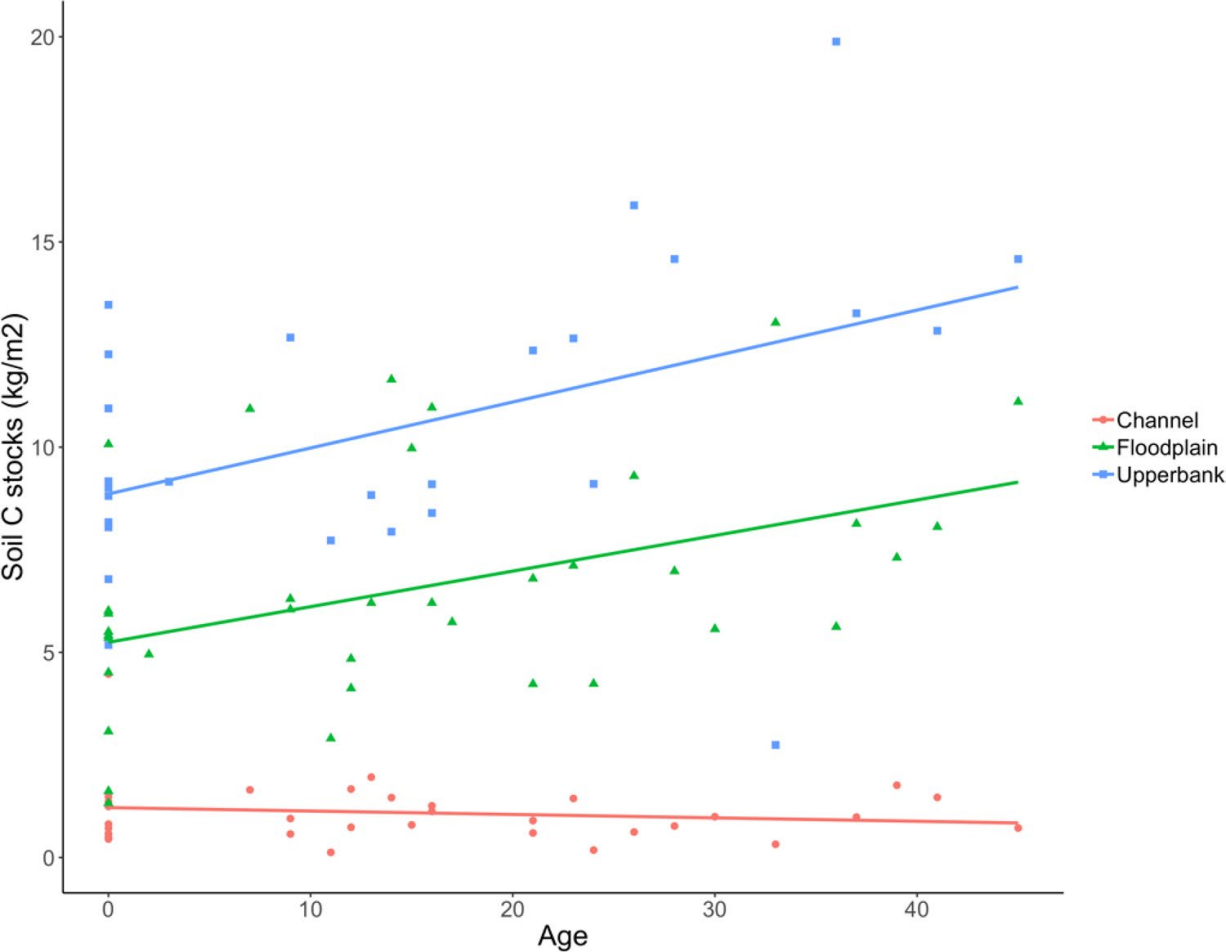
Soil C changes as a function of restoration age for each of three riparian landforms. Soil carbon is expressed in kg m^−2^ to 10 cm depth for the channel and to 50 cm depth for the floodplain and upper bank

We documented increases in riparian carbon storage in soils and woody vegetation as a function of restoration age. Soil carbon measured in the top 50 cm had significant linear relationships with time since revegetation, but only for the floodplain and upper bank landforms (Table 1; Fig. 6); the shallower soils in the active channel exhibited no significant increase or decrease in soil C stocks with increased restoration age. Soil carbon was added at a rate of 0.87 Mg C ha^−1^ year^−1^ on the floodplain (r^2^ = 0.1834, p < 0.01) and 1.12 Mg C ha^−1^ year^−1^ on the upper bank (r^2^ = 0.2144, p < 0.05).

**Table 1:** Regression analysis of soil C (kg m^−2^) and restoration age (year) by landform

Landform	a	b	p	df	r^2^
Channel	1.2159	− 0.008	0.405	31	0.0225
Floodplain	5.2447	0.087	0.009	34	0.1834
Upper bank	8.8594	0.112	0.015	25	0.2144

The components of the regression equation are a = y-intercept and b = regression coefficient for the independent variable “age”

Tree and shrub aboveground biomass carbon accumulation was best described by a logistic (sigmoidal) curve, suggesting that our sites reached canopy closure and maximum biomass over the studied time period. Including both above- and below-ground carbon, the modeled biomass maximum was 187.5 Mg C ha^−1^ in the channel, 279.3 Mg ha^−1^ on the floodplain, and 195.5 Mg ha^−1^ on the upperbank, and successful restoration sites were expected to attain this maximum within two decades (Table 2).

**Table 2:** Modeled carbon accumulation over 50 years of restoration age for riparian buffers on three landforms

Channel					Floodplain					Upper bank				
Age	ABG tree/shrub C	BLG tree/shrub C	Δsoil C	Total	Age	ABG tree/shrub C	BLG tree/shrub C	Δsoil C	Total	Age	ABG tree/shrub C	BLG tree/shrub C	Δsoil C	Total
0	0	0	0	0	0	0	0	0	0	0	0	0	0	0
5	134.65	24.38	0	159.03	5	171.52	30.22	4.34	206.07	5	153.41	27.37	5.60	186.38
10	150.65	28.95	0	179.61	10	214.86	39.68	8.67	263.21	10	186.43	34.98	11.20	232.62
15	152.55	30.55	0	183.10	15	225.82	43.27	13.01	282.09	15	193.54	37.73	16.80	248.07
20	152.78	31.52	0	184.30	20	228.58	45.07	17.34	291.00	20	195.07	39.15	22.40	256.62
25	152.81	32.27	0	185.07	25	229.28	46.26	21.68	297.22	25	195.40	40.14	28.00	263.54
30	152.81	32.89	0	185.70	30	229.46	47.18	26.01	302.66	30	195.47	40.92	33.60	269.99
35	152.81	33.42	0	186.23	35	229.50	47.96	30.35	307.81	35	195.49	41.59	39.20	276.28
40	152.81	33.89	0	186.70	40	229.52	48.63	34.68	312.83	40	195.49	42.18	44.80	282.47
45	152.81	34.31	0	187.12	45	229.52	49.24	39.02	317.77	45	195.49	42.70	50.40	288.59
50	152.81	34.69	0	187.50	50	229.52	49.78	43.36	322.66	50	195.49	43.17	56.00	294.66

Aboveground (ABG) tree/shrub carbon is modeled as a logistic curve, using actual measurements of living biomass in restoration plots aged 1–42 years. Belowground (BLG) tree/shrub carbon is predicted from site age and plot-level live-tree biomass. The change in soil carbon represents the predicted mean value from a linear regression of soil C stock vs. restoration age; the regression was not significant for the channel landform. All values are in Mg C ha^−1^

Using 20-year predicted values for soil and biomass added C, we calculated the added carbon storage that could be expected in Marin County if all rangeland streams had been subjected to streambank restoration measures (Table 3). The total additional carbon in soil and biomass was 284,836 Mg of C or 1,044,399 Mg of CO_2_e (carbon dioxide equivalent). The 95% confidence interval for this estimate ranged from 141,234 to 313,104 Mg C (517,858 to 1,148,048 Mg CO_2_e). The dollar value of this carbon, if priced at the California cap-and-trade program’s May 2019 auction price of $17.45 per ton of carbon dioxide equivalent would be US$ 18.2 million. Expressed in terms of stream linear extent, sequestration amounts to 859.6 Mg of CO_2_e (95% CI 426.2, 944.9) per km of restored stream and a dollar value of $15,000 km^−1^.

**Table 3:** Scale-up to countywide carbon sequestration estimates

Landform	Stream distance (m)	Restorable area (ha)	Mg C/ha	Success %	Total	Low estimate	High estimate
Channel	0–3	207.2	184.30	0.6188	23,630	13,039	28,993
Floodplain	3–12	765.61	291.00	0.7607	169,478	82,939	180,543
Upper bank	12–24	1334.75	256.62	0.2678	91,728	45,256	103,568
Total				Mg	284,836	141,234	313,104
				CO2e	1,044,399	517,858	1,148,048

Restorable area represents grassland land cover within the stream buffer width indicated for the entire 1215-km stream network of Marin County, California. Carbon in Mg C ha^−1^ represents the sum of modeled biomass and soil estimates at 20 years restoration age. Success rate is calculated as the proportion, by area, of the total area attempted that resulted in successful revegetation. Total is the product of restorable area, Mg C ha^−1^, and success rate %; low and high estimates are calculated the same way, but use the endpoints of the 95% confidence intervals for the 20-year point estimates for soil C and biomass C

Restoration had strong effects on soil properties, which also differed systematically among landforms (Table 4). Soil carbon stocks were higher in the restored sites than the unrestored sites (8.89 ± 0.64 vs. 7.72 ± 0.59, F_(1,64.6)_ = 37.6933, p < 0.001), and were also higher on the upper bank than on the floodplain (10.30 ± 0.62 vs. 6.30 ± 0.44; F_(1,44.6)_ = 8.3591, p < .01). Soil N stocks were higher on the upper bank than the floodplain (0.87 ± 0.05 vs. 0.50 ± 0.03, F_(1,45.2)_ = 51.819, p < 0.001) but did not differ significantly by revegetation success (0.70 ± 0.05 for restored, 0.69 ± 0.05 for unrestored, F_(1,66.9)_ = 2.6645, p = 0.1073). A similar pattern was observed for C% and N%. The percentage of soil C was higher in restored sites (1.46 ± 0.09 vs. 1.24 + 0.09 unrestored, F_(1,84)_ = 9.7574, p < 0.01) and higher on the upper bank than the floodplain (1.63 ± 0.09 vs. 1.08 ± 0.07, F(1,84) = 32.2339, p < 0.0001), but the percentage of soil N was only higher on the upper bank (0.14 ± 0.01 vs. 0.08 ± 0.01, F_(1,84)_ = 49.2756, p < 0.0001) and did not differ significantly between restored and unrestored sites (0.11 ± .01 for both, F_(1,84)_ = 3.558, p = 0.062).

**Table 4:** Differences in soil properties between restored and unrestored sites for two riparian landforms

		n	Bulk density	C stock	N stock	Soil C %	Soil N %	C:N	% Labile C	FA:HA
Floodplain	Restored	26	1.18 ± 0.03	7.03 ± 0.51	0.53 ± 0.04	1.21 + 0.09	0.09 ± 0.01	13.4 ± 0.3	.027 ± .001	1.13 ± 0.38
	Unrestored	16	1.38 ± 0.05	5.13 ± 0.72	0.45 ± 0.06	0.85 ± 0.10	0.07 ± 0.01	11.5 ± 0.3	.025 + .001	1.62 ± 0.82
Upper bank	Restored	17	1.43 ± 0.03	11.70 ± 1.13	0.96 ± 0.09	1.83 ± 0.16	0.15 ± 0.01	12.3 ± 0.4	.020 ± .001	0.82 ± 0.37
	Unrestored	25	1.44 ± 0.03	9.39 ± 0.66	0.84 ± 0.06	1.50 ± 0.10	0.13 ± 0.01	11.1 ± 0.2	.021 + .001	1.02 + 0.43

Values are mean ± standard error. Bulk density is expressed in g/cm^3^ and C and N stocks are expressed in kg/m^2^ to 50 cm depth

**Fig. 7:**
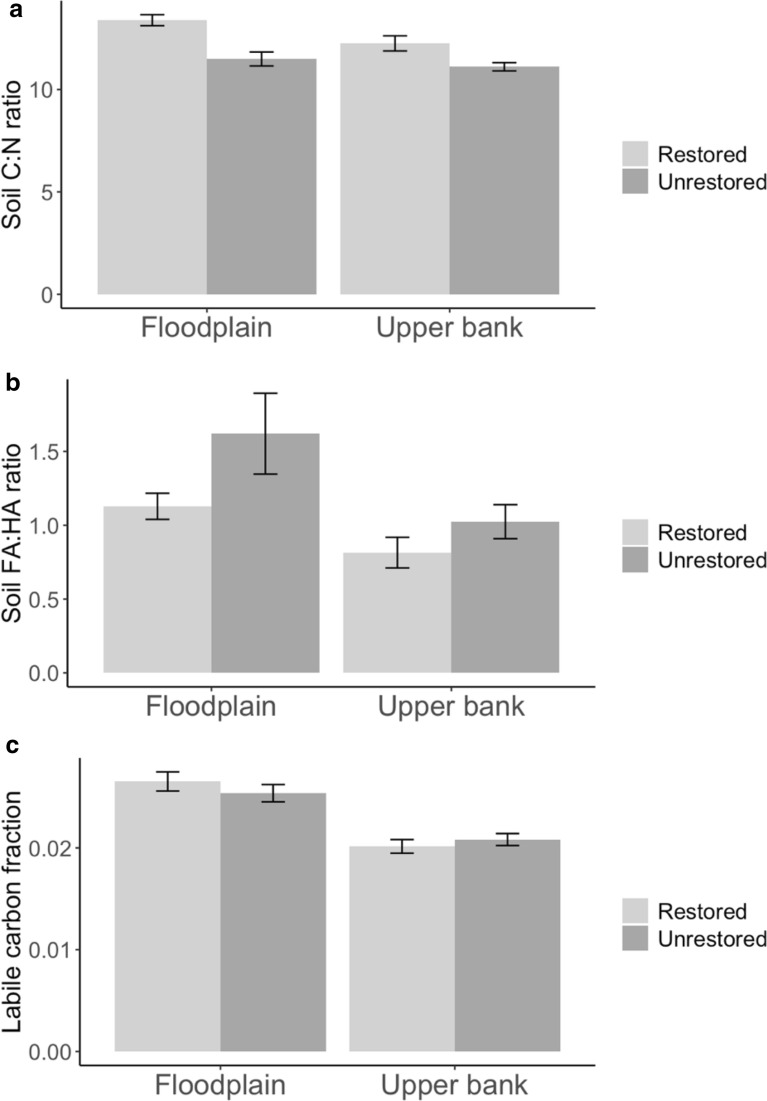
Measures of SOM quality in restored and unrestored sites, by landform. The C:N ratio was higher, and the fulvic acid:humic acid ratio higher, in the restored sites, indicating greater permanence of the soil carbon where riparian revegetation has taken place. Labile carbon was unaffected by restoration status. Landform differences were significant for all three carbon measures. Interactions between landform and restoration status were not significant for any SOM quality measure

The C:N ratio (Fig. 7a) was higher in restored sites than unrestored (12.9 ± 0.2 vs. 11.3 + 0.2, F_(1,80.6)_ = 17.6759, p < 0.0001) and higher on the floodplain than on the upper bank (12.7 ± 0.3 vs. 11.6 ± 0.2, F_(1,42.9)_ = 12.9653, p < 0.001). Another measure of potential differences in SOM quality, the FA:HA ratio (Fig. 7b), was lower in revegetated sites (0.997 ± 0.400 vs. 1.260 ± 0.666, F(1,28.8) = 12.4243, p < 0.01) and was lower on the upper bank as compared to the floodplain (0.923 ± 0.411 vs. 1.290 ± 0.596, F(1,43.4) = 6.9030, p < 0.05). However, the labile carbon fraction (Fig. 7c) did not differ significantly with restoration status (.024 ± .001 restored, .023 ± .001 unrestored; F_(1,84)_ = .0876, p = 0.7680); it differed only by landform, with labile carbon comprising a higher portion of the total carbon on the floodplain compared to the upper bank (.026 ± .001 vs .021 + .0004, F_(1,84)_ = 46.4238, p < 0.0001).

Soil bulk density was governed by a significant interaction between landform and revegetation success, whereby the bulk density was lower in restored sites than unrestored, but this effect was much more pronounced on the floodplain than on the upper bank (F_(1, 58.3)_ = 9.2866, p < 0.01).

**Fig. 8:**
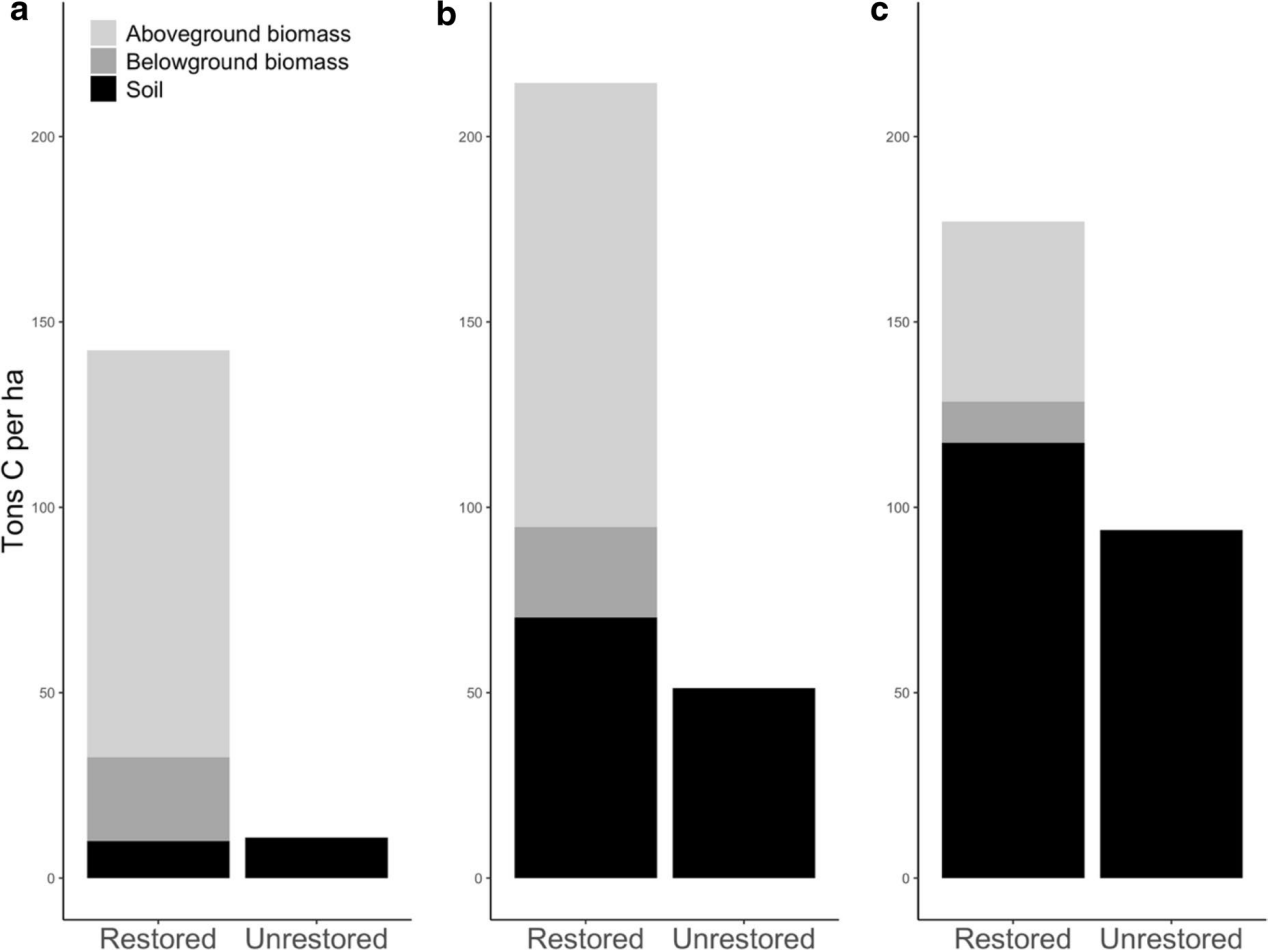
Average values for total carbon, in Mg C ha^−1^, for restored and unrestored sites by landform (**a** channel, **b** floodplain, **c** upper bank). Soil carbon is measured to 10 cm depth for the channel landform and 50 cm depth on the other two landforms. Aboveground and belowground biomass carbon refer only to riparian trees and shrubs; grassland biomass at unrestored sites was not measured

Averaging all sites of all ages together, total carbon varied by landform and was dominated by different pools (Fig. 8). Average soil carbon increased from the channel soils (10.0 ± 1.1 Mg ha^−1^ restored, 11.0 ± 2.2 unrestored) to the floodplain (70.3 ± 5.1 restored, 51.3 ± 7.2 unrestored) to the upper bank (117.4 + 11.3 restored, 93.9 ± 6.6 unrestored). Aboveground and belowground biomass C were zero in the unrestored sites because we only measured C in tree and shrub biomass, not grassland herbaceous biomass. In restored sites, aboveground tree/shrub carbon was similar in the channel (109.7 ± 25.0 Mg ha^−1^) and floodplain (119.8 ± 28.3) but lower on the upper bank (48.6 ± 18.2). Average belowground carbon in restored sites was 22.5 ± 4.8 Mg ha^−1^ in the channel, 24.4 ± 5.3 Mg ha^−1^ on the floodplain, and 11.1 ± 3.8 Mg ha^−1^ on the upper bank.

## Discussion

Revegetating riparian zones to control erosion in northern California rangeland provides a carbon sequestration service that is substantial, valuable, and potentially more stable than carbon stored in comparable rangeland sites that have not undergone restoration. However, the size of this service differs among landforms and is associated with a high degree of uncertainty.

Modeled carbon stocks in mature riparian vegetation ranged from 188 to 279 Mg C per hectare, depending on landform. These estimates are very high compared to the 87.2 Mg C per hectare predicted for restored “willow scrub” riparian forests by CREEC (Carbon in Riparian Ecosystems Estimator for California), a statewide riparian forest carbon calculator [47]. They are higher than predictions for all other California riparian forest types in CREEC, the maximum for riparian woodlands being 245.1 Mg C per hectare. The estimates from our sites also far exceed the expected value of 83 Mg C per hectare for planted riparian vegetation in warm/dry areas of the world, as determined by global meta-analysis [19]. (Data from our sites, in addition to hundreds of other sites, were included in the datasets used to create CREEC and the global meta-analysis.) Our sites may have higher biomass due to the generally cooler, wetter climate in the North Bay region compared to other parts of California. Another possibility is that the high values are an artefact of the small plot size used to take measurements in the narrow channels of our 2nd- and 3rd-order streams. Very small plots may overestimate per-area biomass if they are smaller than the area influenced by the canopy of sampled trees. Therefore, we recommend that our biomass stock values be considered an upper-end estimate of the highest possible sequestration that could be achieved in these sites under optimal conditions. We have also made available our raw data (Additional file 1) to show the size and density of individual stems in each sample plot.

Compared to other mesic forest types in the region, the biomass C in our riparian sites is low. Coast redwood (*Sequoia sempervirens*) forests in our three-county area average 473 ± 61 Mg of C per hectare in live biomass at their canopy maximum, while Douglas-fir (*Pseudotsuga menziesii)* forests average 418 ± 43 Mg [48]. However, compared to the more xeric oak woodlands typical of our rangeland sites, the riparian stocks are more carbon-dense; oak woodland carbon in Marin, Napa, and Sonoma counties averages 89 ± 6 Mg per hectare of C in live tree biomass at canopy maximum (Matzek, unpublished data). Riparian forests may therefore be considered “carbon hotspots” in the dry grassland/woodland habitats of Mediterranean regions, which generally have low annual rates of C sequestration [49].

Carbon in live tree biomass was predicted to accumulate quickly in riparian vegetation, with sites reaching a modeled asymptote for maximum biomass in only two decades. Rates of increase (i.e., coefficient *b* from Eq. 4) ranged from 0.09 to 0.14, depending on landform. Willow scrub forests in CREEC have a predicted rate of increase of 0.17, and values for all riparian vegetation types in CREEC have values of *b* ranging from 0.05 to 0.28, suggesting that our sites are not unusual in this regard. Similarly, the global meta-analysis of riparian forest C found that planted forests reached 90% of their maximum biomass within 15 years for warm/dry regions. Riparian revegetation thus provides a rapid carbon return on planting investment, compared to, for example, Douglas-fir forests in the Pacific Northwest (*b *= .03, Follett et al. [49]).

With respect to soil carbon, a meta-analysis of soil profile studies done in annual grasslands [50] determined that soil C to 50 cm depth in California rangelands averaged 90 ± 5 Mg C per hectare, but that sites with woody vegetation generally had much higher soil C (101 ± 7 Mg C ha^−1^) compared to sites with only nonwoody vegetation (71 ± 6 Mg C ha^−1^). In our sites we found similar trends, but less profound distinctions between woody and non-woody (i.e., restored and unrestored) riparian areas, likely because all of these sites are in more mesic riparian areas. Our sites had an overall average C stock of 83 ± 4 Mg C per hectare, and an average of 89 ± 6 in woody sites vs. 77 ± 6 Mg ha^−1^ in nonwoody sites.

The effects of afforestation on Mediterranean grassland C have been relatively well-studied in Australia, where “environmental carbon plantings” on marginal agricultural land are becoming more widespread [51]. Findings have differed on whether riparian afforestation increases soil C stocks and permanence, with some research studies concluding, as we did, that soil C stocks increase and/or become richer in recalcitrant forms of carbon as a grazed pasture converts to riparian vegetation [52–54]. However, a meta-analysis of Australian sites [55] found that no clear trends in soil C or N stocks, or C:N, could be attributed to tree-planting in Mediterranean-climate grasslands. Studies included in the meta-analysis were quite young (80% ≤ 13 years old) compared to our sites; soil C stock changes can be difficult to detect over short timescales (Smith [56]). Another study performed eight years after restoration showed that changes in soil properties such as total C and N could not be detected although structural changes in the vegetation were observed [57]. The depth at which C stocks are measured also matters; Jackson et al. [58] found that for subhumid (50–100 cm rainfall year^−1^) sites like ours, forest sites had higher proportions of soil C in the upper 20 cm than grassland sites, but below 40 cm the grassland sites generally contained a higher proportion of C.

We observed systematic differences by landform for both biomass and soil C. Tree and shrub biomass C was generally higher in the channel and on the floodplain than on the upper bank. Distance from the water source of the stream may explain lower riparian biomass on the upper bank. Conversely, soil C was highest in the upper bank and lowest in the channel. This is partly due to inherent differences among the landforms in soil depth. We also suspect that we are observing a legacy of erosion, with the more dynamic landforms (channel and floodplain) experiencing greater topsoil loss and consequent C loss than the more stable upper bank landform. This conclusion is bolstered by our observation of higher y-intercepts for our soil carbon vs. age regressions as we move from channel to upper bank, suggesting that at “time zero” before restoration the landforms are already starkly different in C stocks.

Because the carbon in biomass is fated to eventually return to the atmosphere as carbon dioxide, the relative permanence of C stocks can be as important as their size. Belowground organic C typically has longer residence times compared to aboveground C. Residence times of soil organic matter range from tens to thousands of years depending on the quality of the organic matter and the extent to which it is protected from microbial degradation. We used the C:N ratio, FA:HA ratio, and the proportion of labile C in SOM to qualitatively assess differences in source material and the recalcitrance of soil organic matter to microbial decomposition.

The C:N ratio was higher in restored sites, which suggests a change in residue source, or that organic matter is more processed in non-restored sites with lower C:N [59]. Higher C:N in restored sites may reflect an increase in organic matter recalcitrance as low quality organic residues contribute to the SOM pool [60]. The transition from grass to riparian woody vegetation after restoration supports this interpretation. While the difference in C:N was small, it may reflect an expanding trend over time, considering the signature of the large pool of organic matter that was present at time zero.

The ratio of fulvic acids to humic acids was lower in restored sites. These operationally defined substances of humus can be used to evaluate how the environment (climate, vegetation and management) has influenced humus characteristics [61]. Generally, FA are smaller molecular weight compounds with more oxygen-containing functional groups and HA are larger organic molecules. The FA:HA signature may reflect the degree of microbial decomposition of SOM and/or differences in lignin content of the source material [62]. The lower FA:HA values associated with restored sites could reflect a shift to higher amounts of woody organic residues, which by nature decompose slower than annual grass roots. At both landforms, no observable difference in labile C existed between restored and unrestored sites, which is inconsistent with the findings from the other two measures of SOM quality.

The uncertainty surrounding the provision of the C sequestration service by riparian buffer plantings was high, as other work has attested [63]. Our high-end estimate of total C sequestration due to restoration was more than double the low-end estimate. The biomass regression model produced 95% confidence intervals as wide as 120 Mg C ha^−1^ for a 20-year aboveground tree/shrub biomass estimate of 228.6 Mg C ha^−1^, and for soil carbon, the range in the 95% confidence interval for a 20-year estimate covered nearly an order of magnitude, from 4.74 Mg C ha^−1^ to 40.1 Mg C ha^−1^ for upper bank soils. Some of the uncertainty in our regression models was due to variability among sites of similar restoration age. In some sites we deemed “restored,” all or most of the biomass was in weedy shrub species like blackberry, and these sites may represent invasions by less desirable, lower biomass species after the failure of the original riparian plantings. However, we lacked sufficiently specific information about the community composition of the plantings at particular sites to class these sites as failures.

We also observed a highly variable success rate among landforms, with 62% of channel projects (by area) and 76% of floodplain projects meeting the standard of success, but only a 27% success rate on the upper bank. It is possible that some upper bank sites that we classed as failures were in fact never planted, but were only fenced to exclude livestock from the stream and protect plantings in the channel and floodplain. However, in the absence of information needed to distinguish such sites, we made the conservative choice to class them as failures. If the upper bank success rate is inappropriately low, this would in turn greatly underestimate the countywide sequestration potential from our scale-up analysis, because the upper bank landform has the most potentially restorable area.

## Conclusion

Ecosystem services sometimes come in bundles, that is, sets of services that co-occur repeatedly and are associated with the same ecosystem [64]; they may also trade off with each other, i.e., when management efforts to enhance one service end up diminishing another [65]. In our agroecosystems, interventions aimed at restoring a locally relevant ecosystem service (erosion control) simultaneously increased a globally relevant ecosystem service (carbon sequestration). This service is not only substantial but is potentially monetizable, where systems of payment for ecosystem services exist. Despite the relatively small spatial extent of forested riparian systems, revegetation of degraded riparian forests should be included in carbon accounting mechanisms, and considered as a means of diversifying income streams for ranchers and dairy producers.

## Supplementary information

**Additional file 1:** Archived data. Archived data to accompany CBAM-D-19-00053, “Increases in soil and woody biomass carbon stocks as a result of rangeland riparian restoration.” Consists of 4 sheets of data, “individual tree measurements,” “soil carbon by age & success,” “soil properties by landform,” and “biomass C by age and success,” plus a “metadata” sheet explaining all the abbreviations in the other sheets.

## Data Availability

Data from the study are deposited in the open-access archive at https://zenodo.org/record/3941297#.XwuOyi2ZMu8 and as a supplementary file to this paper (Additional file 1)
